# Conservation vs. variation of dinucleotide frequencies across bacterial and archaeal genomes: evolutionary implications

**DOI:** 10.3389/fmicb.2013.00269

**Published:** 2013-09-06

**Authors:** Hang Zhang, Peng Li, Hong-Sheng Zhong, Shang-Hong Zhang

**Affiliations:** Key Laboratory of Gene Engineering of Ministry of Education, and Biotechnology Research Center, Sun Yat-sen UniversityGuangzhou, China

**Keywords:** dinucleotide frequency, compositional analysis, whole-genome sequences, strand symmetry, GC content, primordial genome, origin and evolution of genomes

## Abstract

During the long history of biological evolution, genome structures have undergone enormous changes. Nevertheless, some traits or vestiges of the primordial genome (defined as the most primitive nucleic acid genome for life on earth in this paper) may remain in modern genetic systems. It is of great importance to find these traits or vestiges for the study of the origin and evolution of genomes. As the shorter is a sequence, the less probable it would be modified during genome evolution. And if mutated, it would be easier to reappear at the same site or another site. Consequently, the genomic frequencies of very short nucleotide sequences, such as dinucleotides, would have considerable chances to be conserved during billions of years of evolution. Prokaryotic genomes are very diverse and with a wide range of GC content. Therefore, in order to find traits or vestiges of the primordial genome remained in modern genetic systems, we have studied the characteristics of dinucleotide frequencies across bacterial and archaeal genomes. We analyzed the dinucleotide frequency patterns of the whole-genome sequences from more than 1300 prokaryotic species (bacterial and archaeal genomes available as of December 2012). The results show that the frequencies of the dinucleotides AC, AG, CA, CT, GA, GT, TC, and TG are well-conserved across various genomes, while the frequencies of other dinucleotides vary considerably among species. The dinucleotide frequency conservation/variation pattern seems to correlate with the distributions of dinucleotides throughout a genome and across genomes. Further analysis indicates that the phenomenon would be determined by strand symmetry of genomic sequences (the second parity rule) and GC content variations among genomes. We discussed some possible origins of strand symmetry. And we propose that the phenomenon of frequency conservation of some dinucleotides may provide insights into the genomic composition of the primordial genetic system.

## Introduction

During billions of years of evolution, organic genomes have undergone enormous changes. However, some traits or vestiges of the primordial genome may remain in modern ones. Finding these traits or vestiges is very important for the study of the origin and evolution of genomes (Zhang, 1996). Indeed, the only way to reconstruct ancient genetic systems in the absence of fossil DNA may be the deduction from the comparative analysis of the structures of present-day genomes (see also Birnbaum et al., 2000).

What traits at the genomic level may be regarded as vestiges of the primordial genome? One of our considerations is the genomic frequencies of very short sequences. For a sequence of DNA (or RNA) in a genome, the longer it is, the more probable it would be modified during genome evolution (such as nucleotide substitutions, insertions, or deletions). If a long sequence was changed or disappeared, its original form could hardly emerge again. And the distributions and proportions of long sequences may change greatly during evolution. On the other hand, very short sequences would generally be less influenced by local substitutions, insertions, or deletions. Moreover, if a very short sequence was mutated or disappeared, it would be easier for the sequence to reappear at the same site or at another site in a genome. Therefore, the proportion of a very short sequence, such as a dinucleotide, may have considerable chances to be preserved in a genome during the long history of evolution. In other words, our philosophy suggests that if the frequencies of a dinucleotide in modern genomes are conserved, it would imply that the genomic frequencies of that particular dinucleotide have not changed significantly since the primordial genome formed, hence a vestige of the primordial genome. Based on this assumption, comparative analysis of the characteristics of dinucleotides in the genomes of various organisms may provide insights into the features of the primordial genome as well as the primary genetic information it contained.

For mononucleotides, it has been known that their frequencies vary greatly among species, especially in prokaryotes (Sueoka, 1962). However, that does not preclude the possibility of the conservation of the frequencies of some, if not all, dinucleotides across genomes. Many researches have been done in the field of dinucleotide frequencies even when sequence data were limited (e.g., Nussinov, 1980, 1981, 1984), revealing hierarchies in the frequencies (preferences) of different dinucleotides in natural nucleic acid sequences. With more sequences available, one of the most studied aspects in this field is the characteristics of dinucleotide relative abundances, which access contrasts between the observed dinucleotide frequencies and those expected from the component nucleotide frequencies (Karlin and Burge, 1995). The profiles of relative abundances of dinucleotides in genomic sequences are rather species-specific or taxon-specific (Karlin et al., 1994, 1997). The set of all dinucleotide relative abundance values is even regarded as a genomic signature (Karlin and Burge, 1995). This situation seems in contradiction with our assumption on the conservation of the frequency patterns. However, as assumed above, what we need for the purpose of our study is the occurrence frequencies, which are generally not congruent with the relative abundances (Burge et al., 1992). Moreover, instead of considering the frequencies of all dinucleotides in a genome as a whole, they should be analyzed one by one. Therefore, it is of interest to ascertain if the conservation in terms of dinucleotide occurrence frequencies exists across genomes, or to determine to what extent the frequencies of a dinucleotide vary among species.

With the development of genomics, more and more whole-genome sequences are now available, providing opportunities for the analysis of evolutionary vestiges at the genomic level. Prokaryotic genomes are very diverse and with a wide range of GC content. They are excellent materials for the study of evolutionary genomics. Therefore, for the purpose of finding traits or vestiges of the primordial genome remained in modern genetic systems, we have studied the characteristics of dinucleotide frequencies across archaeal and bacterial genomes. In this paper we analyzed, following our previous preliminary work (Zhang and Yang, 2005), the dinucleotide frequency patterns of the whole-genome sequences from over 1300 prokaryotic species. The results show that the conservation of frequencies of some dinucleotides across genomes does exist. We propose that the frequency conservation patterns would be vestiges of the primordial genome, considering that the phenomenon would depend on strand symmetry of genomic sequences (also called the second parity rule, which is the marked similarity of the frequencies of nucleotides and oligonucleotides to those of their respective reverse complements within single strands of sufficiently long genomic sequences; see Fickett et al., 1992; Prabhu, 1993; Forsdyke and Mortimer, 2000; Qi and Cuticchia, 2001; Baisnée et al., 2002; Zhang and Huang, 2010) and on GC content variations among genomes.

## Materials and methods

### Whole-genome sequences

We downloaded the whole-genome sequence of every species of archaea and bacteria that was available as of December 2012 from the NCBI (via http://www.ncbi.nlm.nih.gov/sites/batchentrez). For the species that have two or more strains or subspecies whose genomes have been sequenced, generally only one was taken from each of them to avoid redundancy (our analysis shows that the choice of strain or subspecies does not influence the validity of the results, data not shown). If there is a difference of more than 1% of genomic GC content between individual strains or subspecies of a species, we selected also the strains or subspecies whose GC content is different from that of others by at least 1% (see also Zhang and Wang, 2012). In total, 133 complete genomes of archaea and 1309 complete genomes of bacteria were analyzed in this study. For the complete list and accession numbers of these genomes, see Supplementary Material 1.

### Calculations of genomic occurrence frequencies of dinucleotides

We counted the number of occurrences of every dinucleotide in each genome. The count was performed by moving the sliding window of 2 nt down the sequence one base at a time. Each chromosome was analyzed separately for genomes with two or more chromosomes, without concatenation. Counts were compiled for each genome. Occurrence frequencies (percentages) were calculated from these counts. The frequencies of dinucleotides containing one or two ambiguous bases were also calculated, but not taken into account because of their usually very small values. In the calculations, only one strand of each genome (the downloaded sequence) was analyzed. Although the choice of strands seems arbitrary, strand symmetry guarantees the validity of the results. In fact, there is little difference in terms of dinucleotide occurrence frequencies in analyzing one strand or another or both strands of a genome (data not shown). All the calculations were performed with computer programs written in Perl.

### Statistical analysis

To determine the level of conservation (or variation) for the frequencies of a dinucleotide across genomes, we employed the correlation/regression analysis. The correlation/regression analysis has been employed to measure the similarity/difference between the frequencies (counts) of an oligonucleotide and its reverse complement (see for example, Prabhu, 1993; Qi and Cuticchia, 2001), and between those of different oligonucleotides (Zhang and Huang, 2010). On the other hand, the χ^2^ test or Fisher's test could not be used in the analysis of frequency conservation/variation because there would always be the significant difference between the counts of any two dinucleotides according to these tests. Even for the counts of a dinucleotide and those of its reverse complement across genomes, they are significantly different in the χ^2^ test (see also Baisnée et al., 2002).

For each dinucleotide, we analyzed the correlation between the observed counts and the expected counts in the genomes studied. The expected count of a dinucleotide in a genome was obtained from the total of all dinucleotide counts of that genome multiplied by the mean frequency (the average frequency of the genomes studied) of that particular dinucleotide. The expected count of a particular dinucleotide in a genome is a hypothesized conserved value in our study. This hypothesized conserved value, if it does exist, could only be estimated from the frequencies (counts) in modern genomes. It seems reasonable to use the average frequency of the genomes studied as an estimating factor. If the frequencies of a dinucleotide are well-conserved across genomes, its observed count and expected count will be very similar in a genome. The expected counts in our study concern occurrence frequencies. They are not the same as the expected frequencies for the calculation of relative abundances (Karlin and Burge, 1995) that are species-specific or taxon-specific (see also Introduction section).

We calculated the Pearson correlation coefficient (*r*). Also, we calculated the slope and the intercept of the best-fitted line for the observed counts vs. the expected counts of the genomes studied. A correlation coefficient and a slope close to 1, and an intercept near the origin would indicate that the frequencies of the dinucleotide concerned are well-conserved across genomes. We converted the correlation coefficients, the slopes and the intercepts to absolute differences from 1, 1 and 0, respectively (the transformed correlation coefficients, the transformed slopes and the transformed intercepts, respectively; see also Zhang and Huang, 2010), so as to measure the levels of frequency conservation/variation for the dinucleotides. We employed the *t*-test to determine the significance of the difference, in terms of transformed correlation coefficients, slopes, or intercepts, between dinucleotides with conserved frequencies and those with frequencies varying considerably among genomes.

## Results

**Figure 1:**
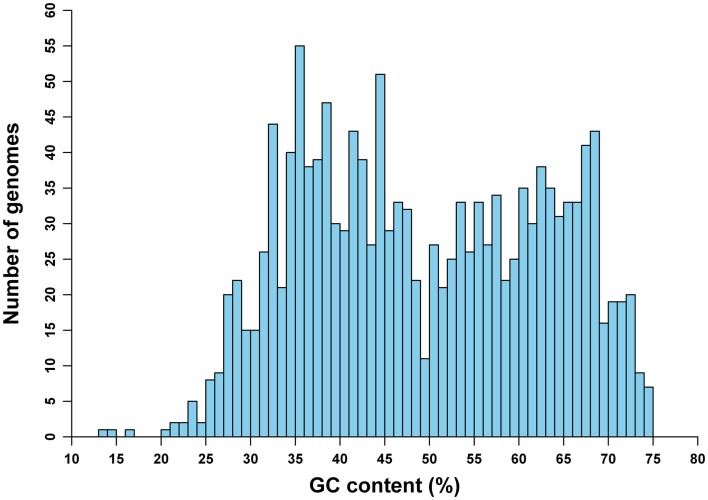
**Distribution of 1442 archaeal and bacterial genomes in terms of GC content**.

The frequencies of mononucleotides vary greatly, from 13.5 to 74.9% in terms of GC content, among genomes in our study (Figure 1). This variation of GC content among genomes is on the whole similar to the distribution of genomic GC content for prokaryotic species with whole-genome sequences available as of December 2008 (Zhang and Wang, 2011). Also, it is comparable to the GC content variation pattern of another more recent study (see Nishida, 2013; the difference, especially for the distribution of genomes with GC content of 50%, may be due to different sampling strategies). This particular distribution pattern may reveal some information about the origin of GC content variations among genomes (see also Discussion).

**Figure 2:**
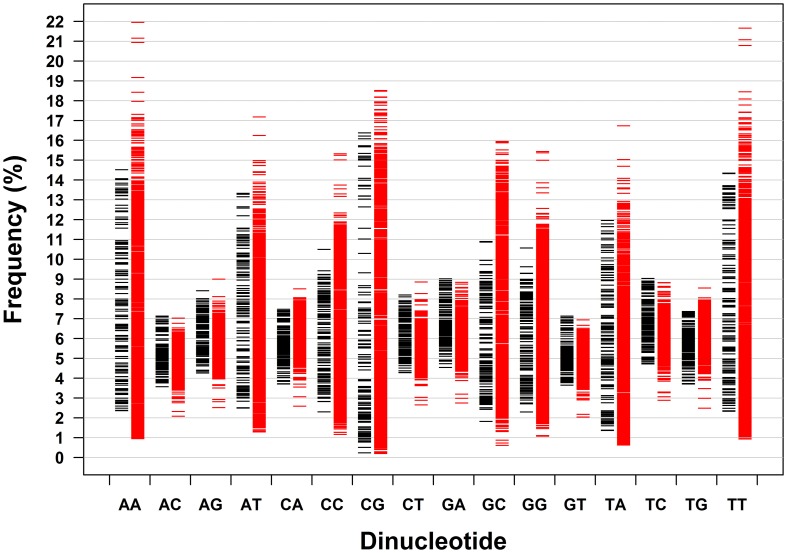
**Dinucleotide frequency distribution patterns of 133 archaeal genomes and 1309 bacterial genomes.** Each genome is represented by a dash (black dash, archaeal genome; red dash, bacterial genome).

Dinucleotides exhibit distinct characteristics from mononucleotides, which apply to archaeal genomes and bacterial genomes taken together and analyzed separately. The distribution pattern of the frequencies of 16 dinucleotides of 133 genomes of archaea and 1309 genomes of bacteria is shown in Figure 2. It is clear that the frequency ranges of the dinucleotides AC, AG, CA, CT, GA, GT, TC, and TG (dinucleotides composed of one strong nucleotide and one weak nucleotide) are much narrower across genomes than those of other dinucleotides (with the mean of each dinucleotide close to the corresponding median, especially for the dinucleotides AC, AG, CA, CT, GA, GT, TC, and TG; Table 1). While most of the genomic frequencies of AC, AG, CA, CT, GA, GT, TC, and TG dinucleotides are clustered around their own means, the distributions of the frequencies of AA, AT, CC, CG, GC, GG, TA, and TT dinucleotides are dispersed throughout most of their respective ranges (see also Supplementary Material 2 for the details of the results). These features are also evident from the statistics such as the standard deviation, the coefficient of variation, the minimum, and the maximum of the dinucleotide frequencies (Table 1). As for the dinucleotide counts, the correlation coefficients between the observed counts and the expected counts of the dinucleotides AC, AG, CA, CT, GA, GT, TC, and TG, respectively, are very close to 1 (*P* < 0.0001); the slopes of the best-fitted lines are also close to 1, and the intercepts are relatively small. For the other eight dinucleotides (AA, AT, CC, CG, GC, GG, TA, and TT), the correlation coefficients may be as low as 0.215 (for archaeal genomes only, *P* = 0.01) and no higher than 0.883 (for all genomes, *P* < 0.0001). Furthermore, the slopes are not close to 1; the intercepts are relatively large. Therefore, all the characteristics described above would indicate that the frequencies of the dinucleotides AC, AG, CA, CT, GA, GT, TC, and TG are well-conserved across genomes; the frequencies of the dinucleotides AA, AT, CC, CG, GC, GG, TA, and TT, on the other hand, vary considerably among genomes. Results of the *t*-test, in terms of the transformed correlation coefficients (*P* < 0.01), the transformed slopes (*P* < 0.0001), and the transformed intercepts (*P* < 0.0001), respectively, could well distinguish the eight frequency-conserved dinucleotides from the other eight frequency-varied dinucleotides (see Supplementary Material 2 for details; it is legitimate to employ the *t*-test because of the normal distribution of the values of the transformed parameter). Actually, given that the frequencies of a dinucleotide are conserved (or varied greatly) across genomes, so are those of its reverse complement, which is consistent with the phenomenon of strand symmetry. Compared with the data of bacterial genomes, it seems that the frequencies of AC, AG, CA, CT, GA, GT, TC, and TG of archaeal genomes are a little less conserved (Table 1 and Supplementary Material 2).

**Table 1:** **Statistical analysis of dinucleotide frequencies and counts across genomes**.

**Dinucleotide**	**Mean (%)**	**Minimum (%)**	**Maximum (%)**	**Median (%)**	***s***	**CV (%)**	***r***	**Slope**	**Intercept**
**ARCHAEA AND BACTERIA, 1442 GENOMES**									
AA	7.87	0.96	21.96	8.04	4.05	51.45	0.41	0.43	179908.90
AC	4.97	2.09	7.14	4.97	0.62	12.52	0.98	0.89	16767.49
AG	5.51	2.53	9.00	5.42	0.75	13.62	0.97	1.06	−6844.52
AT	6.95	1.30	17.19	7.00	2.90	41.75	0.57	0.66	104193.71
CA	6.14	2.60	8.51	6.19	0.80	13.03	0.96	0.96	7721.61
CC	6.18	1.17	15.33	5.98	2.66	43.01	0.88	0.52	92839.19
CG	6.83	0.21	18.52	5.74	4.57	66.86	0.83	0.36	140063.00
CT	5.50	2.66	8.86	5.41	0.75	13.61	0.97	1.06	−6701.52
GA	6.11	2.75	9.02	6.05	0.84	13.80	0.97	0.91	17739.81
GC	7.40	0.62	15.95	6.70	3.59	48.56	0.87	0.46	123839.40
GG	6.19	1.08	15.44	6.01	2.65	42.86	0.88	0.52	92689.23
GT	4.97	2.05	7.14	4.97	0.62	12.45	0.98	0.89	16503.86
TA	5.18	0.63	16.74	4.96	3.31	63.89	0.25	0.24	149866.06
TC	6.09	2.89	9.04	6.04	0.84	13.77	0.98	0.90	17959.29
TG	6.14	2.50	8.56	6.19	0.79	12.93	0.96	0.96	7153.56
TT	7.87	0.94	21.67	7.97	4.04	51.38	0.40	0.43	179813.37
**ARCHAEA, 133 GENOMES**									
AA	7.65	2.37	14.52	7.19	3.51	45.95	0.38	0.27	128522.43
AC	5.16	3.58	7.14	5.07	0.85	16.47	0.93	0.70	33104.11
AG	6.19	4.27	8.42	6.23	0.99	15.96	0.89	0.99	3523.57
AT	7.20	2.50	13.33	7.34	2.96	41.13	0.44	0.35	108152.38
CA	5.69	3.71	7.49	5.62	0.88	15.38	0.86	0.85	19881.17
CC	6.12	2.31	10.50	6.12	2.00	32.68	0.81	0.53	62294.28
CG	5.81	0.24	16.38	4.43	4.55	78.36	0.68	0.20	100952.24
CT	6.18	4.29	8.21	6.17	1.00	16.15	0.89	0.99	3666.17
GA	6.82	4.55	9.02	6.73	1.09	15.95	0.94	0.70	42773.60
GC	5.70	1.83	10.90	5.18	2.32	40.66	0.77	0.42	72428.50
GG	6.12	2.30	10.57	6.15	2.01	32.93	0.81	0.53	62871.92
GT	5.16	3.66	7.14	5.07	0.86	16.63	0.92	0.70	33267.64
TA	6.04	1.37	11.96	5.88	2.87	47.51	0.21	0.17	115554.00
TC	6.82	4.73	9.04	6.75	1.09	16.02	0.94	0.70	42890.44
TG	5.68	3.71	7.37	5.66	0.86	15.06	0.87	0.87	18430.04
TT	7.64	2.34	14.35	7.19	3.49	45.72	0.38	0.27	128202.44
**BACTERIA, 1309 GENOMES**									
AA	7.90	0.96	21.96	8.14	4.10	51.94	0.38	0.40	193623.16
AC	4.95	2.09	7.04	4.97	0.59	11.94	0.98	0.88	17895.43
AG	5.44	2.53	9.00	5.38	0.68	12.58	0.97	1.04	−4322.86
AT	6.92	1.30	17.19	6.94	2.89	41.81	0.56	0.64	111892.59
CA	6.18	2.60	8.51	6.23	0.78	12.59	0.96	0.97	5242.11
CC	6.19	1.17	15.33	5.97	2.72	43.91	0.88	0.52	97709.58
CG	6.94	0.21	18.52	5.87	4.56	65.72	0.83	0.36	147307.16
CT	5.43	2.66	8.86	5.37	0.68	12.54	0.97	1.04	−4188.99
GA	6.03	2.75	8.84	6.01	0.78	12.89	0.98	0.89	20085.44
GC	7.57	0.62	15.95	6.87	3.65	48.27	0.87	0.47	130605.23
GG	6.20	1.08	15.44	5.98	2.71	43.73	0.88	0.52	97545.85
GT	4.96	2.05	6.95	4.97	0.59	11.85	0.98	0.88	17577.20
TA	5.09	0.63	16.74	4.77	3.34	65.58	0.25	0.22	155384.24
TC	6.02	2.89	8.83	6.00	0.77	12.81	0.98	0.89	20320.74
TG	6.19	2.50	8.56	6.24	0.77	12.49	0.96	0.98	4537.72
TT	7.89	0.94	21.67	8.07	4.09	51.89	0.38	0.40	193543.39

*Mean, minimum, maximum, median, s (standard deviation), and CV (coefficient of variation) are statistics for dinucleotide frequencies; r is the Pearson correlation coefficient between the observed counts and the expected counts (P = 0.01 for TA dinucleotide of archaeal genomes; P < 0.0001 for all others); slope and intercept are the two parameters of the best-fitted line for the observed counts vs. the expected counts*.

As our results show, there is a general correlation between the observed counts and the expected counts of a dinucleotide in the genomes studied, a correlation observed even for dinucleotides whose frequencies are not conserved across genomes. This general correlation is mainly due to the usual trend that the observed counts of a dinucleotide increase with genome sizes, hence somewhat trivial. Therefore, what is important and interesting in our results is the finding that the observed counts and the expected counts of some dinucleotides are very highly correlated. This special correlation is due to frequency conservation across genomes of the dinucleotides concerned.

The correlation/regression analysis and other statistics indicate that the frequencies of the dinucleotides AC, AG, CA, CT, GA, GT, TC, and TG are well-conserved across genomes, while the frequencies of the dinucleotides AA, AT, CC, CG, GC, GG, TA, and TT vary considerably among species. Though our results concern only prokaryotic genomes, actually they apply also to eukaryotic genomes (for a preliminary analysis, see Zhang and Huang, 2008). Moreover, the frequency conservation/variation patterns are phylogeny- and taxon-independent (see also data in Supplementary Material 2). Therefore, the conservation of the frequencies of some dinucleotides across genomes is a ubiquitous phenomenon. In fact, the conservation of the frequencies of the dinucleotides AC, AG, CA, CT, GA, GT, TC, and TG is more obvious than that of any trinucleotide or higher-order oligonucleotide (see Zhang and Huang, 2008). The fact that only half of the 16 dinucleotides are well-conserved in terms of genomic frequencies is in concordance with the situation that the genomic frequencies of mononucleotides vary greatly among genomes.

## Discussion

The conservation of the frequencies of some dinucleotides we reported is universal in modern archaeal genomes and bacterial genomes. Also, it is found in eukaryotic genomes, even if they have a large proportion of non-coding sequences. There would be two alternative approaches to explain the existence of these universal features: considering them as evolutionary convergences or, alternatively, as vestiges of the primordial genome. The “convergence” approach has to reveal the selective advantages or mutation pressures leading to the universal pattern, which are not apparent (see below). Therefore, we would try the “vestige” approach. No matter whether these compositional features are due to structural constraints or other factors on nucleic acid sequences, some of the constraints or factors might exist from the very beginning of genome evolution. In this sense, it is possible to regard the universal compositional features as evolutionary vestiges rather than convergences.

An early study indicates that there are significant correlations between genomic libraries in terms of tetranucleotide frequency distribution, suggesting an overall correlation of frequency profiles of short nucleotides among genomes (Rogerson, 1991). Our finding shows that the frequency conservation involves especially some dinucleotides. Causes for this phenomenon may include: (1) patterns of distributions of dinucleotides throughout a genome and across genomes; and (2) probabilities of occurrences of dinucleotides set by strand symmetry. It has been shown that genome inhomogeneity is determined mainly by AA, TT, GG, CC, AT, TA, GC, and CG dinucleotides (consisting of two strong nucleotides or two weak nucleotides), which are closely associated with polyW and polyS tracts (W and S stand for weak nucleotides and strong nucleotides, respectively; Kozhukhin and Pevzner, 1991). That implies the distribution of any one of the other eight dinucleotides (SW and WS dinucleotides, i.e., AC, AG, CA, CT, GA, GT, TC, and TG) in a genome is rather homogeneous. Also, the distributions of oligonucleotides containing similar and especially the same numbers of the strong and weak nucleotides, but no CG or TA dinucleotide, are the most uniform in six representative genomes (yet the authors considered their distributions not informative; Häring and Kypr, 1999). The results of our analysis are consistent with these distribution patterns. Therefore, one reason for the frequency conservation across genomes of some but not all dinucleotides would be that only the distributions of the frequency-conserved dinucleotides are quite uniform throughout a genome and across genomes.

More importantly, if the probability of occurrences of a dinucleotide is fixed to a certain range by the frequencies of its component nucleotides (which themselves follow the rule of strand symmetry), the variation of its actual frequencies will also be limited. For example, in our analyzed prokaryotic genomes, the AT content varies from 25.1 to 86.5%; the GC content varies from 13.5 to 74.9%. Under the regime of strand symmetry, the expected frequencies of AA, AT, TA, and TT dinucleotides may vary from 1.6% (with the frequencies of A and T being both approximately 12.6%) to 18.7% (with the frequencies of A and T being both approximately 43.3%); those of CC, CG, GC, and GG dinucleotides from 0.5% (with the frequencies of C and G being both approximately 6.8%) to 14.0% (with the frequencies of C and G being both approximately 37.5%). With strand symmetry and GC content variation in the same way, the expected frequencies of AC, AG, CA, CT, GA, GT, TC, and TG dinucleotides will range only from 2.9% (AT content being 86.5%, GC content being 13.5%) to 6.3% (both AT content and GC content being 50.0%). Therefore, strand symmetry would contribute to the frequency conservation of the dinucleotides AC, AG, CA, CT, GA, GT, TC, and TG across genomes, while the frequency variations of the dinucleotides AA, AT, CC, CG, GC, GG, TA, and TT would largely depend on GC content variations among genomes.

The current hypotheses about the origins of strand symmetry can actually be classified into four categories: (1) selection of stem-loop structures (Forsdyke, 1995a,b); (2) no strand biases for mutation and selection (Lobry and Lobry, 1999); (3) strand inversion/inverted transposition (Fickett et al., 1992; Albrecht-Buehler, 2006); and (4) original trait of the primordial genome (Zhang and Huang, 2008, 2010). However, the contribution of stem-loop potential of single-stranded DNA to strand symmetry would be very limited (Zhang and Huang, 2010). In addition, it seems that the “no strand bias” hypothesis can account for first-order symmetry (strand symmetry for mononucleotides) only (see also Albrecht-Buehler, 2006). Finally, whether the inversions/inverted transpositions have been numerous and widespread enough in the course of evolution to lead to strand symmetry as found in modern genomes is a challenging issue.

Alternatively, we have suggested that strand symmetry would probably exist from the very beginning of genome evolution (Zhang and Huang, 2008, 2010). In fact, it has been proposed that the biological diversity in the primordial biosphere (the number of species of DNA or RNA macromolecules capable of self-replicating on a large scale) would be very low due to competitive exclusion, and that repeats of a certain species of self-replicating macromolecules made up the most primitive genomes (Zhang, 1998). When these macromolecules formed repeated sequences with sufficiently abundant repeating units by connecting one after another, it would most probably result in approximately equal amounts of forward repeats and their reverse repeats (for single-stranded RNA, the reverse repeating units would be the complementary sequence of the forward repeats), leading naturally to strand symmetry (Zhang and Huang, 2008). If the phenomenon of strand symmetry in modern genomes is a vestige of the primordial genome, so must the frequency conservation pattern linked to it. In that case, no matter what the GC content of the primordial genome was, its dinucleotides AC, AG, CA, CT, GA, GT, TC, and TG were with frequencies not considerably different from the corresponding ones in modern genomes. The mechanisms for maintaining strand symmetry (see Nussinov, 1982; Lobry and Lobry, 1999; Sanchez and Jose, 2002; Albrecht-Buehler, 2006) would also help maintain the frequency conservation pattern. As for the origin of the frequency variations of the dinucleotides AA, AT, CC, CG, GC, GG, TA, and TT, it must be linked to the origin of GC content variations among genomes. Our preliminary analysis would indicate that there is the possibility that GC content variations among genomes would also be primitive traits (our unpublished data). In this sense, the dinucleotide frequency conservation/variation patterns described in this paper would have their origin dating back to the very early period of genome evolution.

From this study and our related work, it would be reasonable to conclude that the information revealed from modern genome structures in terms of dinucleotide frequencies could be helpful for the reconstruction of the primordial genome as well as for the further understanding of the pattern of genome evolution. And the understanding of the primary genetic information contained in the primordial genome would certainly shed light on the origin of genomes, and even on the origin of life.

## Author contributions

Hang Zhang wrote the computer programs and analyzed data; Peng Li analyzed data; Hong-Sheng Zhong help wrote the computer programs; Shang-Hong Zhang designed the study, analyzed data, and wrote the paper.

### Conflict of interest statement

The authors declare that the research was conducted in the absence of any commercial or financial relationships that could be construed as a potential conflict of interest.

## Supplementary material

Supplementary material 1 **List of archaeal and bacterial genomes analyzed**. Excel spreadsheet containing the list and related information of the archaeal and bacterial genomes studied.

Supplementary material 2 **Dinucleotide frequencies of the archaeal and bacterial genomes studied**. Excel spreadsheet showing the details of the dinucleotide frequencies and statistical analysis for the archaeal and bacterial genomes studied.
